# Preconception Expanded Carrier Screening: A Discourse Analysis of Dutch Webpages

**DOI:** 10.3390/healthcare11101511

**Published:** 2023-05-22

**Authors:** Sofia Morberg Jämterud, Anke Snoek

**Affiliations:** 1 Department of Thematic Studies, Technology and Social Change, Linköping University, 581 85 Linköping, Sweden; 2 Department of Ethics, Law and Medical Humanities, Amsterdam University Medical Center, De Boelelaan 1117, 1081 HV Amsterdam, The Netherlands

**Keywords:** preconception expanded carrier screening, epistemology, ethics, rationalities, discourse analysis, genetics

## Abstract

Preconception expanded carrier screening (PECS) informs prospective parents about the risk of conceiving a child with a heritable genetic condition. PECS will also, for many, become an important screening test, and websites will likely play a vital role in providing information on this practice. The aim of this article is to examine rationalities in the information on PECS on Dutch websites. The method used is multimodal critical discourse analysis. This method allows an examination of norms and assumptions in the descriptions, as well as of the positions that are discursively made available. The data consist of publicly available material on websites from two genetics departments in the Netherlands. In the results, we present the three main discourses and subject positions that were identified: risk and the couple as possible mediators of severe conditions; the focus on scientific facts and rational conceivers; and severity of the conditions and the responsible couple. In this study, we highlight the importance of acknowledging the interrelation between epistemology and ethics in the discourse on PECS. Finally, it is claimed that the focus on scientific facts in information on PECS risks making existential and ethical dilemmas and choices invisible.

## 1. Introduction

Expanded carrier screening is a practice that informs prospective parents about the risk of conceiving a child with a heritable genetic condition [1,2,3,4]. Previously, carrier screening was limited to screening for a single or a few diseases among high-risk groups [5,6]. However, technological advances have made expanded carrier screening possible, and it is now possible to screen for several hundred genetic conditions regardless of ancestry [7,8]. The range of genetic conditions that are screened for differs between health care providers globally [9]. For example, one provider in the Netherlands screens for 50 conditions [9], while a screening project in Australia (Mackenzie’s Mission) has selected a larger gene list and screens for around 750 conditions [10,11]. Certain professional bodies suggest that offering expanded carrier screening, both as pre-pregnancy and prenatal carrier screening, can be important as a means of enhancing reproductive autonomy [12,13], but also point to the importance of genetic counselling for couples undergoing this form of screening test [13,14]. Studies on the public’s attitudes toward *preconception* expanded carrier screening (PECS) show that there is a great deal of interest in this form of screening [15,16] and that the severity of disorders influences attitudes on the acceptability of participating in this form of screening [17]. Studies of participants in PECS testing have also shown the advantages of offering the test before conception, since this can decrease the risk of anxiety among couples [18]. One of the main aims of offering expanded carrier screening is to enhance reproductive autonomy [19,20,21], and it has been suggested that when this screening test is offered before conception, it allows prospective parents many different reproductive choices, in contrast to when offered as prenatal screening [22]. However, other aims in line with public health ethics have recently been suggested as being important in the implementation of this form of carrier screening [23].

In this study, we examine the information about PECS on the webpages of the two genetics departments in the Netherlands that offer the test to the general public. The method used is critical discourse analysis. Websites are an important platform for providing information to the public and are also utilised by the public for information on health. One example is that in 2020, 55% of citizens in the European Union aged 16–74 used the internet as a source of health-related information [24]. Other studies have shown that websites play an important role for information on genetic testing in relation to reproduction [25].

Websites on medical issues most often aim at presenting information in a neutral and non-directive way. However, information that is presented always offers a choice, such as regarding which facts are chosen (or not chosen) to be presented and the way in which the information is presented. Hence, webpages can be seen to declare rationalities. The aim of this article is to examine rationalities in the information on PECS on Dutch websites. Such an examination is important since information on webpages can play a role in shaping views on PECS not just at the individual level, but also at the socio-cultural level.

Since expanded carrier screening is fairly new, few studies have examined information on this form of screening. Previous studies have explored how expanded carrier screening has been presented to the public in the mass media [26,27] and have also included analyses of online marketing by health care providers of expanded carrier screening [28]. However, to our knowledge, no discourse analysis aiming to examine rationalities in the information provided on webpages about PECS has been conducted.

## 2. Materials and Methods

### 2.1. Critical Discourse Analysis

Discourse, in a broad sense, concerns how meaning is constructed through language. Discourse can be defined as, “[…] a particular way of talking about and understanding the world (or an aspect of the world)” [29] (p. 1). In this study, a critical discourse analysis was used; this is an approach that examines discourse as a social practice, hence the focus on the context of language and the use of language in relation to social, cultural and political formations. In using critical discourse analysis as a method, we therefore agree with the assumption that not only does language and how we talk convey neutral information about the world and social relations, but the way that language is used also contributes to their shaping and creation [29,30]. Critical discourse analysis, therefore, allows for an analysis of text as a social process [31] and is useful since it focuses on critical examination of ideologies, norms and assumptions within a specific discourse. Furthermore, the connection between knowledge production and social action is regarded as important, since discourses create and uphold a certain worldview, and within this view, certain forms of social action will be restrained or unthinkable, while other forms of social action will be seen as natural [29,30]. Hence, the frames for social action and worldview are closely tied together in discourse. This also ties into the analysis of “subject positions”. A subject position is produced through discourse, “[…] discourses construct *subjects* as well as objects and, as a result, make available positions within networks of meaning that speakers can take up (as well as place others within)” [32] (p. 132). Certain positions and ways of acting are thus discursively made available by the descriptions and constructions. Hence, the focus and interest of the analysis is on which positions are discursively constructed; the ways of acting, responding and speaking that are made possible; and also which limits are created [32]. Furthermore, a discourse sets limits on what is regarded as meaningful. The subject position is therefore constructed in relation to what is understood as logical and meaningful [29].

A multimodal approach was chosen, meaning that text in combination with visual images were analysed [30,31]. The strength of such an approach is that the analysis can include meaning-making that is produced in different forms of communication. Visual images can reinforce certain messages or operate in a complementary way in relation to written text [31].

In the discourse analysis, the chosen webpages were closely and thoroughly read, and each page was analysed with a focus on four areas: 1, presentation of genetic knowledge; 2, carriership; 3, choice; and 4, benefits and risks of the test. Genetic knowledge and carriership were chosen because these two areas are central in the practice of PECS. Choice and presentations of benefits and risks of the screening test were chosen based on previous literature studies of ethical perspectives on PECS.

The webpages were read by AS in Dutch and in a translated version by SMJ. In the analysis, the reading focused on the content, use of language and which norms and values were represented. We also analysed the accompanying visual images, with a focus on which ideas were communicated and which norms and values were represented [30]. In the study, we have strived to be transparent in our presentation of the interpretations made and which theoretical lens has been adopted, in order for the reader to be able to critically discuss the results [29].

The analysis was conducted between March and October 2022.

### 2.2. Setting and Material

The choice was made to analyse website information on PECS in the Netherlands because it has been available to anyone wishing to test for a number of years, and not only to high-risk groups. The primary corpus of material analysed in the article consists of publicly available material from the websites of Amsterdam Universitair Medische Centra (AUMC) and of Universitair Medisch Centrum Groningen (UMCG), which host the two genetics departments that offer PECS tests. Hence, the websites are informational websites of regional health care systems and not commercial websites. Our paper focuses on PECS as an offer to the general public, and not for at-risk groups. However, AUMC targets both groups. AUMC has a longer history of offering PECS to at-risk groups, but then extended its offer to the general public as well. UMCG is situated in the city of Groningen in the far north of the Netherlands. It is considered a more rural area. UMCG developed a PECS offer for the general population in 2016.

The genetics departments at AUMC and UMCG differ in how many conditions they screen for and how they offer the test. AUMC offers screening for 50 autosomal recessive conditions and UMCG for 70 autosomal recessive conditions, such as cystic fibrosis and SMA (spinal muscular atrophy). The fact that the conditions are autosomal recessive means that both parents must be carriers of the genetic condition in order for the child to be at risk of being born with it. Most people do not know that they are carriers, since it is possible to be a carrier without having any symptoms. In this way, the genetic condition will only become known to the individual through a DNA test. As previously described, expanded carrier screening can also be offered in early pregnancy, but this study focuses on text presenting the test as given preconceptually.

At AUMC, a couple can order a test directly from the genetics department through the university website. The partners are tested sequentially, so if the first partner tests negative, there is no need to test the second partner. This option is less expensive when the first test is negative. At UMCG, the test is offered through general practitioners. If a couple are interested in taking a test, they contact the general practitioner directly for pre-test counselling and the test. The test is then processed at UMCG. UMCG tests partners simultaneously, and does not provide individual test results.

**Figure 1:**
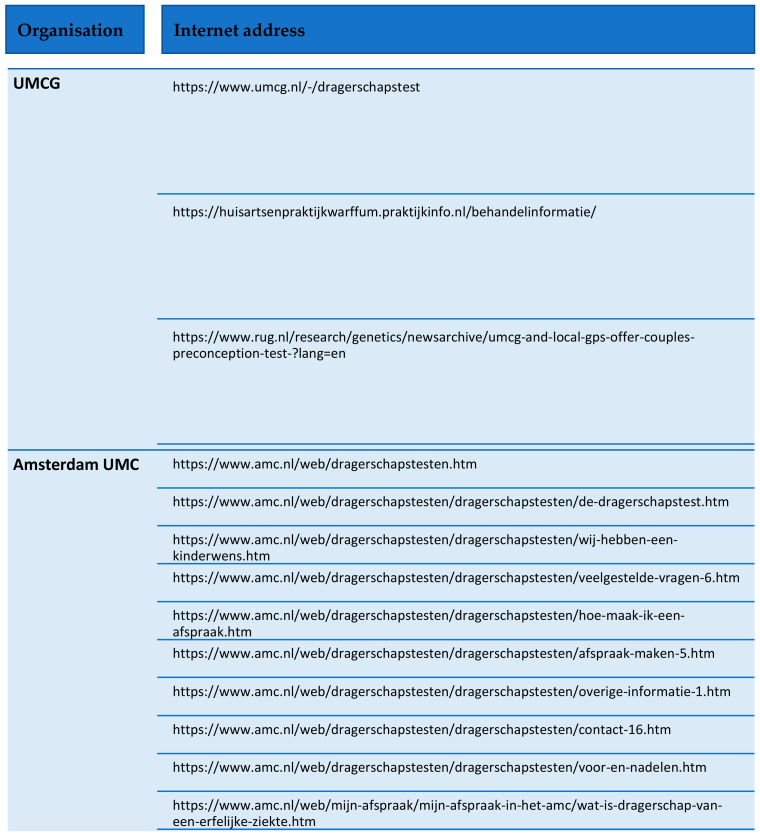
Data collection—webpages.

The corpus comprised 13 webpages in total (Figure 1). The two sites have different designs, with one requiring the use of subsequent links to gain access to information (AUMC), while the other (UMCG) keeps all its information on one page. This results in an uneven set of webpages between the two organisations. They also vary in terms of how much visual information is presented, with AUMC making use of images, in contrast to UMCG, which is text-based. Neither of the websites make use of video material. The descriptions on the webpages are adapted for people without genetics expertise.

## 3. Results

### 3.1. The Discourse on Risk and the Couple as Possible Mediators of Severe Conditions

The UMCG website starts with the following statement, “Everyone carries various abnormalities in their genes that can lead to disease in their children. We call this carrier status” [33]. The literal translation from Dutch would be, “Everyone is a carrier” (Iedereen is drager). Hence, the first message is that we are all carriers of abnormalities [afwijkingen] in our genes. AUMC has a similar, but slightly toned-down message: “Everyone is a carrier of a predisposition to one or more diseases. So being a carrier is nothing special” [34]. The second message on the UMCG website relates to our unawareness of this, “Usually, you do not know whether you are a carrier of a certain condition, because often it doesn’t affect you” [33]. It is stated that being healthy yourself is not a good indicator of whether you have abnormalities in your genes that can affect your child:

“That is why you can unexpectedly have a child with a hereditary disease, although you are not ill yourself”.

[33]

Words such as “unexpectedly” (onverwacht) draw attention to the surprise factor that there are no visible indicators that signal that one could give birth to a child with a hereditary condition. Therefore, there is a cluster of messages saying that all are carriers of a predisposition to one or more diseases, we are mostly unaware of this, and that being healthy is no guarantee that a joint child will not be affected. This creates what we describe as a balanced tone of caution—it is not an alarming tone, but still a caution that one ought to be aware of the risks. Here, it is worth remembering that the information on the webpages is directed at couples in the general population who do not necessarily have any previous indication of being carriers, for example through known hereditary conditions in the family. Hence, a cautious tone can motivate people to act, and becomes a catalyst for taking the test and becoming aware of risks.

On the webpages, there is extensive explanatory work on risk: information about risk, who is considered to be at risk, what the risk concerns are and the calculation of risk. Risk becomes the rationale for the importance of offering PECS. Risk is presented as a, “[…] calculable understanding of genetic risk that can be obtained only through genetic testing” [35] (p. 342), and the language of calculation is used at both UMCG and AUMC. As noted in previous research, statistics and calculation are specific forms of communication which are not neutral and, “[…] imply an abstract mathematical universe” [36] (p. 67). This form of communication might not be understood by all, which can be a reason why simplified statistics are often presented, as is also the case in the webpages examined. On the webpages, the inheritance of autosomal recessive disease is explained in a language of calculation, as this is knowledge that provides an indication that a couple could be a possible “carrier couple”. AUMC explains:

“If both partners are carriers of the same hereditary disease (carrier couple), there is a 1 in 4 (25%) chance in each pregnancy that the child will have the disease. It does not matter whether it is a girl or a boy. A carrier couple also has a 3 in 4 (75%) chance in each pregnancy that their child will not have the disease. However, there is a 1 in 2 (50%) chance that a child will also be a carrier”.

[37]

This text is also accompanied by an illustration of the inheritance of autosomal recessive disease and shows set patterns of risk. The image shows a male and a female who are carriers. It illustrates that they both have a predisposition to the disease (illustrated in red) as well as a healthy gene (illustrated in white). The image then shows how their offspring has four possibilities: healthy child (25%), carrier child (25%), carrier child (25%) and affected child (25%). The discourse described, through its subtly cautious tone, creates a position of the couple as possible mediators of severe conditions and as a possible threat to any future child’s health—while at the same time stating that there is a 75% chance that the child will not have the disease. The information can shape understandings of reproduction and of the importance of considering genetic compatibility before conceiving. Furthermore, this knowledge about how autosomal recessive disease is inherited can be seen as shaping and reframing who is considered to be at risk, i.e., future children conceived by persons in the general population where there is no previous indication of being carriers of genetic disease, such as belonging to a high-risk group.

### 3.2. The Focus on Scientific Facts and Rational Conceivers

There is a strong focus on the websites on genetic knowledge and gaining knowledge about carrier status. “The carrier test is intended for all couples who want to have children and who want to know whether their future child has an increased risk of a serious hereditary disease” [33]. Scientific facts are presented, such as explanations on how autosomal recessive diseases are inherited and calculable forms of risk. The information is written in comprehensible language that indicates that a certain genetic illiteracy is presumed. On the AUMC webpages, which use visuals in their information, some images accentuate the scientific status of the information.

In one of the images, a person is working in a laboratory surrounded by laboratory equipment, wearing a lab coat and latex gloves, indicating that they are involved in scientific practice. Such visuals also have a cultural significance, since they emphasise robust and trustworthy science [30]. Furthermore, the fact that the information originates from a highly credible scientific source—genetics departments—resonates with ideals of validity and trustworthiness.

The webpages’ focus on scientific facts can be understood as aiming to present the PECS test in a value-neutral way. This can be compared with genetic counselling, where one central principle has been non-directiveness, aiming to present facts and information neutrally, for example regarding genetic conditions and the likelihood of a genetic condition running in a family, in order for an individual to make an autonomous choice based on their own values [38,39]. However, a consequence of this is that facts are presented and information is given in a non-directive way; however, existential and ethical aspects of taking a PECS test are absent. One example of this is the presentation of options that a couple have if a test result comes back positive. UMCG explains:

“

- Get pregnant naturally and have a prenatal test during pregnancy. If it turns out that the child has inherited the disease, you can choose to terminate the pregnancy or prepare for a child with the disease
- Get pregnant naturally, do nothing and accept the risk of disease
- Get pregnant via test-tube fertilisation and have an embryo selection done beforehand with IVF/ICSI. Only embryos without the disease are returned to the womb
- Fertilisation using donor sperm or a donor egg”.

[33]

The alternatives are stated without further explanation about what these alternatives could mean for the couple on an emotional/existential level—for example, in relation to IVF or an abortion. Discourse marked by the prevalence of scientific facts creates a discursive position for a couple which we name “rational conceivers”. Couples are positioned as making rational choices based on scientific knowledge—such as facts about the inheritance of autosomal recessive diseases and calculated risks—while existential, ethical and emotional quandaries or reactions are largely absent in the website discourse. While medical science and a screening test can provide knowledge, give partial information and reduce some insecurities in advance, the genetic knowledge provided can have other consequences. Framing the message as, “Do you want to know?” and providing only scientific knowledge ignores the existential, ideological and value aspects that underlie this question. A similarity can be drawn to other forms of gene testing, where similar detachment has been discerned. The description of gene testing as an uncomplicated test preventing disease and providing individuals with knowledge can stand in the way of other descriptions, as noted by Solbrække et al., “[…] although gene testing provides modern subjects with an opportunity to foresee their biological destiny, it undoubtedly also comes with difficult existential dilemmas and choices” [40] (p. 91). In addition, the scientific knowledge looks straightforward and calculable, while the potential existential aspects often follow a different rationale.

### 3.3. Severity of the Conditions and the Responsible Couple

A key message on the webpages is that the conditions that the PECS test screens for are “severe”, and the test is presented as a solution for couples who want to gain knowledge of any possible increased risk of conceiving a child with any of these conditions. We claim that this discourse can create a subject position of the “responsible couple”.

On the webpages, the information about which conditions are screened for is presented differently. AUMC provides a list of the conditions, but no additional information is provided on these, while UMCG does not state which conditions it screens for. However, both describe certain general criteria. UMCG explains on the Dutch webpages:

“These are serious diseases that are congenital or start at a very young age. These diseases are difficult to treat, or untreatable and can lead to death at a young age”.

[33]

The following description is provided on the UMCG English webpage:

“These diseases lead to serious physical problems and intellectual disabilities, sometimes with severe pain, are difficult or impossible to treat, and can lead to early death”.

[41]

AUMC states:

“

- the disease starts in childhood
- the child has a (severe) intellectual disability
- the child suffers from a (severe) disability and/or is in pain
- the child may die at a young age or have a significantly shortened life expectancy
- the child is expected to have to visit the hospital for treatment on a regular basis
- the condition cannot be cured”.

[42]

The webpages seek to underline a message, i.e., the severity of these conditions, and terms such as “severe” (ernstige, zware) recur on the webpages of both UMCG and AUMC. As can be seen, the webpages point to similar criteria, and the picture this provides is that a child born with any of these conditions would have to live with great pain; that there is very little hope for a future without illness, since the conditions are difficult to treat or non-treatable; and that the child and the family would have to endure living with the knowledge that these conditions can lead to the early death of the child. The websites’ focus on the severity of the conditions creates a picture of a life for a potential child and family that parents would want to avoid for their child, since shielding a child from danger and suffering is at the heart of parental responsibility. The webpages present the PECS test as a solution to such a situation—an opportunity to use a technology that can prevent the birth of a child with a severe condition—and this message is highlighted. It is stated:

“Thanks to this test [the carrier test], couples who want to have children can find out whether they have an increased risk of having a child with one of these diseases”.

[43]

The accompanying image is of two people forming a heart shape with their hands, signalling that they are a couple. The caption to the image reads, “We want to have children” [43]. It can also be noted that the image only shows a couple, and children do not appear in this or any of the other images. This draws attention to the possibility of taking a PECS test before conception and gaining knowledge about potential risks.

The webpages’ focus on severe conditions, in combination with the presentation of the PECS test as a solution to gain knowledge about increased risk, creates a discursive position of the responsible couple. A responsible couple uses the technology to gain knowledge about their genetic disposition in order to prevent any future child from having to live with a severe disease. Furthermore, this focus can create a position of being irresponsible if one does not take a test. This also connects to what has been noted by Swoboda in stating that new reproductive technology creates a certain, “[…] model of reproduction against which prospective parents’ actions are judged” [25] (p. 231). This underlines the moral dimension in the position of “responsible/irresponsible”. It also ties into a larger discursive shift where individuals increasingly become responsible for their own health [44], and as an extension in this case, couples can be seen as responsible for the future health of a potential child.

Another important aspect to consider is how the discourse of this test positions the couple as responsible when they plan pregnancies ahead, since this becomes vital if they are to avoid the risk of genetic disease in a potential child. The temporal aspect of this test and its association with active pregnancy planning reflects a specific perception of life and social values that previous research has connected to discussions of social class [40]. Research has pointed to the planning of parenthood as connected to a middle-class view of life where pregnancies ought to be planned in relation to other aspects in a life chronology, such as education and career steps [45]. “Taking it as it comes” or “just getting pregnant” does not fit into that cultural perspective. Instead, the working class is often stereotyped as accidentally becoming pregnant, which is also perceived negatively [46]. This dichotomy comes to the fore in discourse on PECS, where responsibility becomes connected with being informed, planning pregnancies and foreseeing risk, while irresponsibility could be understood as connected to unplanned pregnancy, entangled with the risk of potential illness and ignorance.

## 4. Discussion

The aim of this article is to examine rationalities in the information on PECS on Dutch websites. We have analysed three main discourses and subject positions, namely: risk and couples as possible mediators of severe conditions; the focus on scientific facts and rational conceivers; and severity of the conditions and the responsible couple.

In the results, the interrelation of epistemology and ethics can be discerned. The way that knowledge is presented can have ethical implications; one example from our result is the description of severe conditions which discursively position the couple as ‘the responsible couple’. This interrelation will be discussed in more detail.

As shown, the webpages focus on presenting scientific medical knowledge. Knowledge regarded as scientific is often connected to being objective, and within feminist science and technology studies, the meaning of objectivity has been discussed at length. Donna Haraway points out that objectivity, often understood as neutral and “from nowhere”, can disguise a very particular position, which becomes universal. Such an understanding of objectivity risks making other positions invalid. Haraway coined the term “situated knowledge” as a way of underlining that we see and understand objects from a certain perspective; we are situated subjects and our positionality shapes our knowledge. From Haraway’s perspective, “objects of knowledge” are not passive or separate in relation to the “knowing subjects.” Instead, she points to the interconnectedness between the two [47]. However, knowledge being situated does not necessarily mean that all knowledge is only subjective [48].

In adapting Haraway’s analytic lens to our result, we want to pinpoint that the interpretation of the meaning of “severe” conditions is not neutral, but knowledge produced from a specific position. Furthermore, the presentation of “severe conditions” on the webpages does not open up alternative views on what severity could comprise. There is, of course, no doubt that the illnesses are severe, but in what way? Previous research has shown that severity of conditions is an important factor when people consider PECS [17]. However, studies have also shown the difficulty for participants to discern classifications of severity, partly due to phenotype variations. Furthermore, participants differ regarding what they consider to be “severe” [49]. As has been noted by Ilana Löwy, there is a difference between predicting that a child will have a specific genetic disease and the severity of the expression of the disease. She exemplifies this with SMA. Löwy points to the fact that even though a test of the genetic compatibility of a couple can state a probability of the child being born with a genetic disease, it cannot predict how severely it will be affected. As in the case with SMA, this disease can come in different forms—from mild to severe. Hence, a life with SMA can vary according to severity [50]. These aspects that Löwy points to—which also underline a broader spectrum of complexity—are not brought up in the information on the analysed websites. The websites describe the conditions as severe and state the criteria. They do not go into details about the different forms or expressions. Hence, what severe diseases are is not unambiguous, but can encompass greater complexity than a binary view of severe or not severe. The reason this is important to acknowledge is that one specific position and interpretation of the meaning of a severe condition risks becoming naturalised as a fact in couples’ reflexive deliberations. This can also have ethical implications in relation to how couples understand that they ought to act. Studies have shown that a reason for considering PECS is linked to how parental responsibility is understood [51], and even though the main aim for offering PECS is to enhance reproductive autonomy, parental responsibility has been suggested as an additional aim [19]. In this study, we pointed to how the presentation of severe conditions *discursively* can position the couple as responsible. More inclusive webpage information, which opens up a broader understanding of severity and which acknowledges the complexity on this matter, would allow for variations in the interpretation of what a “responsible act” could be.

The discussion of the description of severity of conditions also aligns with the discussion about how discourse on the use of technology is not neutral. Instead, the aim of offering a specific technology can be connected to underlying norms and values that the use of the technology will help the users to achieve, which can tie into larger cultural understandings and values [52]. To point to the conditions being severe is an idea that can rely on specific norms, such as an interpretation of compassion in which it is ethical to minimise avoidable suffering in the individual case, and to contribute to genetic selection against harmful conditions [53,54]. However, this interpretation can be articulated at the expense of other interpretations of life with genetic disease, such as narratives that depict stigmatisation, and narratives in which life with a genetic condition is not experienced as unbearable [55]. Since previous research has shown that couples have different reasons in deciding whether to take or not take a PECS test—such as being prepared [56] or the burden of knowledge [57]—information on webpages could provide a more complex picture of PECS. This would, in turn, provide persons with a broader understanding of PECS and thus enable persons to form their own views about PECS that are in line with their values and norms.

Hence, based on this discussion and Haraway’s theoretical contribution regarding the interrelation between epistemology and ethics, we suggest that it is important to open up the information for different epistemological interpretations, hence acknowledging and addressing complexity. We have exemplified this with the presentations of conditions as severe which discursively position the couple as responsible. However, the interrelation is also applicable to other elements of the information.

In the results, we also pointed out the focus on scientific facts and the discursive position of the couple as rational conceivers. With such a focus, ethical and existential dilemmas and choices risk being made invisible, even though presenting information in a neutral way can come with the best intentions.

Information within health care often tries to communicate medical issues in a simple way for clarity, but the result can be that complexities risk being made invisible. Furthermore, research has noted that existential issues such as fear, death and vulnerability are rarely included in information on health care issues such as screening or genetic testing [58]. Researchers within the field of medical humanities have recognised that medical science has sometimes overlooked the importance of providing both medical and clinical knowledge and acknowledging that humans are meaning-making subjects trying to make sense of medical practices. Some have described this as an “epistemic gap” [59]. We acknowledge a similar epistemic gap in relation to the information on PECS. The language in the information on PECS is focused on scientific facts, which can overlook that such knowledge can evoke existential feelings and questions. This focus on scientific facts could also signal that there is a correct answer, based on facts, to whether it is right or wrong to take a test [58]. However, following a medical humanities approach, the suggestion would be to also include writings which acknowledge humans as meaning-making subjects and take into account that information about these forms of screening tests can evoke existential questions that cannot be answered with scientific facts. It should be noted that the genetics departments offer genetic counselling to couples who have decided to take a test, taking seriously that people need to make choices in alignment with their views of life, which also includes engaging with couples in meaning-making activity. The significance of counselling before a PECS test has also been highlighted in research [60]. However, also making visible in information on webpages that there can be existential, normative sides of the decision to test or not to test could provide a broader perspective and be a way of bridging what has been described as an “epistemic gap” [59].

### 4.1. Implications for Policy

Even though many prefer information on PECS directly from health care providers or secondly in the form of written material [61], webpages have shown to be an important source for information on issues of health [24]. Hence, there are good reasons to presume that webpages will be an important source for information on PECS for the general public. In this study, it is suggested that a more complex picture of the practice of PECS should be included in informational material, such as the complexity of severity of genetic disease. Furthermore, it is also important that the information on webpages provides information about possible existential and normative aspects of the decision to test or not to test.

### 4.2. Limitations of the Study

The material in this study is only from the Netherlands, so the quantity of the material is limited. It is possible that webpages informing on PECS in other countries could provide different forms of information. However, we found it to be of interest to reflect on the development of this rather new practice and how it is offered to the public when it is in a seminal state. Since this is a discourse analysis, the findings in the study are not generalisable. A limitation is also that the analysis of webpages was performed during a specific period of time, and that images as well as text may have changed.

### 4.3. Future Research Perspectives

To further examine the issues raised in our study, future studies could focus on which information couples taking the test would appreciate to find on websites. The design of this study could be qualitative or include more participatory action research. Participatory action research could bring together web designers, clinical geneticists, GPs who offer the test and couples who have taken the test.

## 5. Conclusions

As PECS is a relatively new practice targeting young couples, most of them will probably seek information online. PECS will also, for many, become an important screening test, and websites will likely play a vital role in providing information on this practice. Bearing this in mind, it is important to also examine discourse on PECS on websites. In line with other medical websites, the websites studied here presented the information in a factual, technical manner. We distinguished three discourses and subject positions in our analysis: (1) risk and the couple as possible mediators of severe conditions; (2) the focus on scientific facts and rational conceivers; and (3) severity of the conditions and the responsible couple. The aim of medical websites to present information in a non-normative way does not do justice to the existential upheaval these tests often cause patients, and the focus on scientific facts risks making existential and ethical dilemmas and choices invisible. Although factually there is nothing wrong with how the websites present information, persons could get help to be better prepared for the existential dimension of the test. In this study, the interrelation between epistemology and ethics in discourse on PECS is highlighted. It is suggested that a more complex and broader description of PECS on websites (for example, regarding the meaning of “severity” of conditions) could enable persons to form their own views about PECS that are in line with their values and norms.

## Data Availability

All data available in publication.
